# Acute Lymphoblastic Leukaemia Cells Impair Dendritic Cell and Macrophage Differentiation: Role of BMP4

**DOI:** 10.3390/cells8070722

**Published:** 2019-07-14

**Authors:** Jaris Valencia, Lidia M. Fernández-Sevilla, Alberto Fraile-Ramos, Rosa Sacedón, Eva Jiménez, Angeles Vicente, Alberto Varas

**Affiliations:** Department of Cell Biology, Faculty of Medicine, Complutense University, 28040 Madrid, Spain

**Keywords:** acute lymphoblastic leukaemia, BMP4, dendritic cells, macrophages, tumour immune microenvironment

## Abstract

Dendritic cells and macrophages are common components of the tumour immune microenvironment and can contribute to immune suppression in both solid and haematological cancers. The Bone Morphogenetic Protein (BMP) pathway has been reported to be involved in cancer, and more recently in leukaemia development and progression. In the present study, we analyse whether acute lymphoblastic leukaemia (ALL) cells can affect the differentiation of dendritic cells and macrophages and the involvement of BMP pathway in the process. We show that ALL cells produce BMP4 and that conditioned media from ALL cells promote the generation of dendritic cells with immunosuppressive features and skew M1-like macrophage polarization towards a less pro-inflammatory phenotype. Likewise, BMP4 overexpression in ALL cells potentiates their ability to induce immunosuppressive dendritic cells and favours the generation of M2-like macrophages with pro-tumoral features. These results suggest that BMP4 is in part responsible for the alterations in dendritic cell and macrophage differentiation produced by ALL cells.

## 1. Introduction

It is currently known that tumour microenvironment influences tumorigenesis, cancer progression and metastasis [1,2]. The tumour microenvironment consists of non-cellular and cellular components including the extracellular matrix, stromal cells such as fibroblastic and endothelial cells, and immune cells such as lymphocytes and myeloid cells [3,4,5]. Dendritic cells (DCs) and macrophages (MØs) are commonly found infiltrating solid and haematological tumours and carrying out their anti-tumoural activities at the initial stages of tumour development. However, as the tumour progresses, signals derived from tumour cells and their microenvironment impair the differentiation, maturation and function of DCs and MØs transforming them into immunosuppressive cell types with pro-tumoural functions [6,7,8,9]. Manipulation of tumour-derived factors affecting tumour-associated DCs and MØs therefore constitute a new strategy for treating human solid and haematological malignancies [10,11,12,13].

Acute lymphoblastic leukaemia (ALL) is a malignant haematological disorder of the early lymphoid progenitors, occurring in all age groups. In children, ALL is the most common type of haematological malignancy, mainly affecting early B cell precursors [14,15]. Alterations in the numbers of different immune cells have been reported in childhood ALL [16,17,18,19], but to our knowledge, there is no data available about the involvement of DCs and MØs in paediatric ALL progression.

Bone Morphogenetic Proteins (BMPs) are multifunctional growth factors which constitute a subgroup of the TGF-β superfamily. BMPs participate in the regulation of cell proliferation, differentiation and death in embryonic and adult tissues [20], and are also involved in cancer, where they can function as both tumour suppressors and tumour-promoting factors [21,22]. The role of the BMP signalling pathway in leukaemia progression is beginning to be revealed [23,24], and particularly for ALL, the increased production of BMP4 by bone marrow mesenchymal stem cells at diagnosis and the up-regulated expression of some BMP ligands by ALL blasts support a role for BMP signalling in this haematological disorder [25,26,27].

In this study, we provide evidence that ALL cells are able to impair the differentiation of DCs and MØs from monocytes, and BMP4 seem to mediate part of the effects induced by ALL cells.

## 2. Materials and Methods

### 2.1. ALL Cell Lines

The B cell precursor leukaemia cell line Nalm-6 (ACC128) was obtained from DSMZ (German Collections of Microorganisms and Cell Culture) and maintained at a density of 1 × 10^6^ cells/mL in RPMI-1640 with 10% FBS and antibiotics (all from Lonza).

To generate a stable cell line overexpressing BMP4, Nalm-6 cells were transduced with lentiviruses expressing an empty construct (control Nalm-6) or a construct encoding BMP4 (BMP-4-transduced Nalm-6). BMP4 mRNAs and protein expression levels were quantified by quantitative real-time PCR and ELISA, respectively. Cells with high levels of transduction and gene expression were used for further experiments. Lentivirus vectors for BMP4 overexpression were prepared with pMDG, p8.91 and an empty lentiviral expression plasmid or a lentiviral expression plasmid encoding BMP4 as described [28]. BMP4 in pCMV-SPORT6 (Clone Id: 4399276; Termofisher) was transferred via pDONR201 (Invitrogen) to the lentiviral plasmid pLNT-SFFV-WPRE-Gateway (obtained through Dr Peter van der Sluijs from Dr Adrian Thrasher, University College London, UK). Recombinant lentiviruses were prepared as described above. BMP4 cDNA was verified by dye termination sequencing.

Conditioned media were obtained by culturing control and BMP-4 transduced Nalm-6 cells at a density of 5 × 10^6^ cells/mL for 48 h.

### 2.2. Monocyte Isolation and Culture 

Buffy coats from healthy donors (Centro de Transfusión de la Comunidad de Madrid, Spain) were used to obtain peripheral blood mononuclear cells by density gradient centrifugation with Lymphocytes Isolation Solution (Rafer). Monocytes were isolated by positive magnetic separation using CD14 immunomagnetic beads (Miltenyi Biotec). To generate DCs, CD14^+^ cells (10^6^/mL) were cultured for 5–6 days in RPMI-1640 with 10% FBS plus 20 ng/mL rhGM-CSF and 20 ng/mL rhIL-4 (Invitrogen, Termo Fisher Scientific), and half of the medium was replaced by fresh medium with cytokines every 2 days. CD14^+^ monocytes (5 × 10^5^/mL) were also cultured for 3–5 days with 5 ng/mL rhGM-CSF or 10 ng/mL of rhM-CSF (ImmunoTools GmbH) to obtain M1 or M2 MØs, respectively, and in both cases the same concentration of cytokines was added every 2 days. To study the effects of leukaemia-derived soluble factors, monocytes were differentiated to DCs and M1 MØs in the presence of 50% conditioned media from control and BMP4-transduced Nalm-6 cells.

### 2.3. PCR Analysis

RNA isolation was performed using Absolutely RNA Microprep Kit (Stratagene Cloning Systems, Agilent Technologies), including a DNase I digestion step, as recommended by the supplier, to avoid genomic DNA contamination. Total cDNA was synthesized using High Capacity cDNA Reverse Transcription Kit (Applyed Biosystems, Thermo Fisher Scientific), according to the supplier’s instructions, and then used as target in the PCR amplifications. Real-time PCR was performed using pre-designed TaqMan probes from Applied Biosystems (Table 1).

All PCR reactions were set in duplicate using the TaqMan Gene Expression Master Mix (Applied Biosystems, Thermo Fisher Scientific) according to the manufacturer’s instructions. The amplifications, detections and analyses were performed in a 7.900HT Fast Real-time PCR System (Centro de Genómica, Complutense University, Madrid, Spain). The ΔCt method was used for normalization to GNB2L1 mRNA. 

### 2.4. Flow Cytometry

The following mAbs conjugated to FITC/Alexa 488, PE, PE-Cy5 or APC/Alexa 647 were used for flow cytometry analysis: CD1a (HI149), CD3 (HIT3a), CD4 (OKT4), CD8 (RPA-T8), CD14 (47-3D6) and CD163 (GH/S1) from ImmunoStep, BioLegend and BD Biosciences. Immunofluorescence stainings were carried out by incubating cells in PBS containing 1% FBS and 0.1% NaN_3_ in the presence of saturating amounts of fluorochrome-conjugated mAbs for 30 min at 4 °C. To avoid binding of antibodies to Fc receptors, cells were pre-incubated for 5 min at 4 °C with FcR Blocking Reagent, following supplier´s instructions (Miltenyi Biotec). All analyses were conducted in a FACSCalibur flow cytometer (BD Biosciences) from the Centro de Citometría y Microscopía de Fluorescencia (Complutense University, Madrid, Spain)

### 2.5. Mixed Lymphocyte Reaction Assays

DCs differentiated in the absence or presence of Nalm-6-derived conditioned media were cultured overnight with 50 ng/mL LPS and then used as stimulators for allogeneic CD3^+^ T cells isolated with nylon wool columns from buffy coats (DC:T cell ratio, 1:10). Lymphocytes were labelled with 5 μM CFSE (Sigma-Spain) to determine their proliferative response by the CFSE dilution method after 5–6 days of culture.

### 2.6. BMP4 and Cytokine Measurements

Culture supernatants from mixed lymphocyte reaction assays were harvested and the levels of IFN-γ were assayed by ELISA (R&D Systems). The production of BMP4 was determined using an ELISA kit from R&D Systems and following the manufacturer´s instructions.

### 2.7. Statistical Analysis 

The Mann–Whitney test was used to compare differences. Values of *p* ≤ 0.05 (*), *p* ≤ 0.01 (**) and *p* ≤ 0.001 (***) were considered to be statistically significant.

## 3. Results

### 3.1. ALL Cells Induce the Generation of DCs with Immunosuppressive Features 

**Figure 1:**
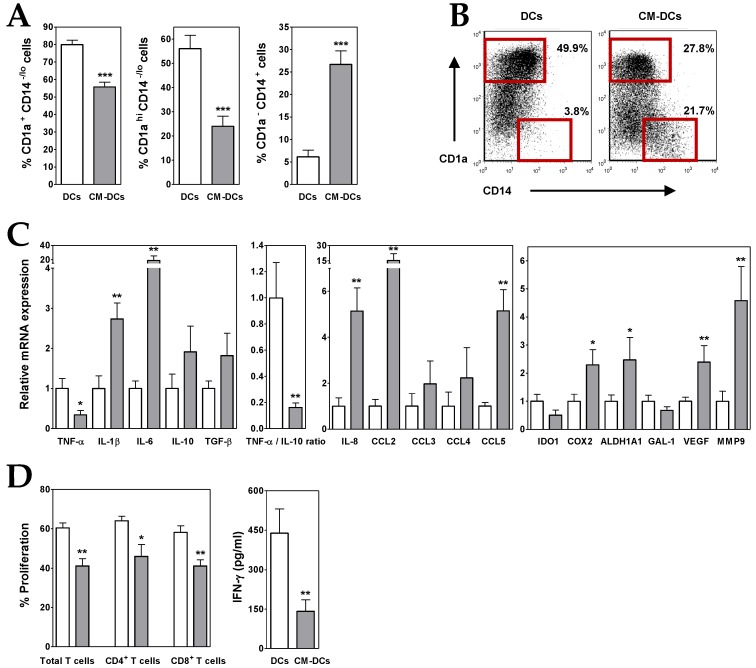
ALL cells alter the differentiation of dendritic cells (DCs). (**A**) Percentages of CD1a^+^ CD14^−/lo^, CD1a^hi^ CD14^−/lo^ and CD1a^−^ CD14^+^ cells recovered after 5–6 days of culture in the absence (white bars; DCs) or presence (grey bars; CM-DCs) of conditioned media from ALL cells. Data represent the mean ± SEM of 12 to 15 independent experiments. (**B**) Representative dot plots showing CD14 versus CD1a expression. Percentages of CD1a^hi^ CD14^−/lo^ and CD1a^−^ CD14^+^ cell populations, delimited by red gates, are shown. (**C**) Real-time PCR quantification of mRNA levels in DCs differentiated from monocytes in the absence (white bars) or presence (grey bars) of conditioned media from ALL cells. Relative mRNA expression was calculated by dividing all individual data by the mean expression in control DCs. Results represent the mean ± SEM of five to seven independent experiments. (**D**) Histograms show the percentages of proliferating CD4^+^ and CD8^+^ T cells, gated on the CD3^+^ cell population and calculated by the CFSE dilution method in mixed lymphocyte reaction assays. Data are the mean ± SEM of seven independent experiments. Supernatants from DC/T cell co-cultures were harvested at day 5-6 and the amount of IFN-γ was quantified by ELISA. Data are the mean ± SEM of three to six independent experiments. Asterisks represent statistically significant differences between DCs and CM-DCs (* *p* ≤ 0.05, ** *p* ≤ 0.01 and *** *p* ≤ 0.001; by Mann–Whitney test).

Human peripheral blood monocytes were induced to differentiate into DCs in the presence or absence of conditioned media (CM) from ALL cells. During this differentiation process, monocytes downregulated CD14 and upregulated CD1a, so that after 5 days of culture under control conditions CD1a^+^ CD14^−/lo^ cells constituted the main cell population, and moreover, most of them expressed high levels of CD1a (Figure 1A,B). However, the presence of ALL-derived CM induced a reduction in the proportion of total CD1a^+^ CD14^−/lo^ cells, mainly affecting the terminally differentiated CD1a^hi^ CD14^−/lo^ DCs (~30% and 60%, respectively), and concomitantly a notable increase in the proportion of CD1a^−^ CD14^+^ cells (Figure 1A,B). In addition, a reduced expression of HLA-DR was also observed (Supplementary Figure S1A). These results suggested that one or more factors contained in ALL-derived CM altered the differentiation of DCs. Interestingly, Nalm-6 ALL cells expressed BMP4, but not other related BMP ligands such as BMP2, BMP6 or BMP7 (Table 2), and neutralization of BMP4 in ALL-derived CM reduced (by 40–50%) the accumulation of CD1a^−^ CD14^+^ cells and increased the generation of CD1a^hi^ CD14^−/lo^ DCs (data not shown; manuscript in preparation).

A further analysis of DCs generated in the presence of ALL-derived CM (CM-DCs) showed that these cells exhibited an immunosuppressive profile. In CM-DCs, transcription of TNF-α was repressed whereas IL-10 expression was increased, which resulted in a drastic decrease of TNF-α/IL-10 expression ratio (~80–90%) (Figure 1C). An enhanced expression of other cytokines, such as IL-1β and TGF-β, as well as chemokines, such as IL-8, CCL3, CCL4 and CCL5, was also detected in CM-DCs (2- to 5-fold increases), being IL-6 and CCL2 the ones that underwent the highest increase (19- and 15-fold increase, respectively) (Figure 1C). Likewise, CM-DCs showed an upregulated expression (2- to 5-fold increases) of pro-tumoural factors such as vascular endothelial growth factor (VEGF), matrix metalloproteinase-9 (MMP9), and cyclooxygenase-2 (COX2) and aldehyde dehydrogenase 1A1 (ALDH1A), both enzymes involved in biosynthesis of prostaglandins and retinoic acid, respectively (Figure 1C). However, the expression of indoleamine 2,3-dioxygenase-1 (IDO1) enzyme and the carbohydrate-binding protein galectin-1 (GAL-1), both with immunosuppressive activity, was reduced in DCs differentiated in the presence of ALL-derived CM (Figure 1C).

To analyse the capacity of ALL cells to affect DC functionality, CM-DCs were co-cultured with allogeneic CD3^+^ T lymphocytes. In correlation with the above results, the mixed lymphocyte reaction assays revealed that CM-DCs were able to stimulate T cell proliferative responses less efficiently than control DCs (Supplementary Figure S2). Figure 1D shows that the proliferative responses induced by CM-DCs were on average reduced by 30%, affecting both CD4^+^ and CD8^+^ T cells. In addition, levels of IFN-γ secreted by activated T cells were severely reduced in the presence of CM-DCs (Figure 1D). 

### 3.2. BMP4 Overexpression Potentiates the Ability of ALL Cells to Induce Immunosuppressive DCs

**Figure 2:**
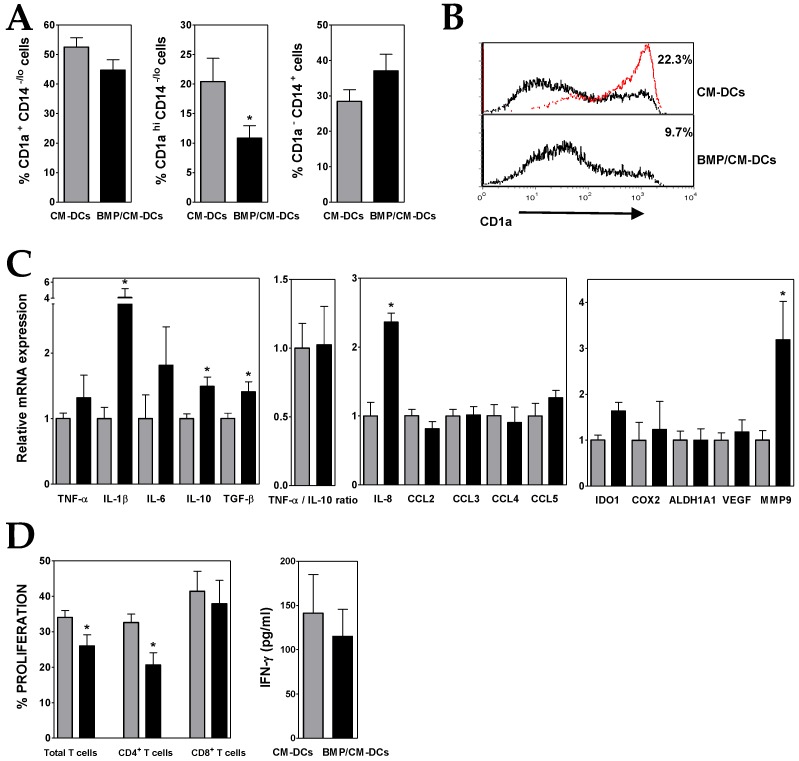
BMP4-overexpressing ALL cells exhibit a higher capacity to generate immunosuppressive DCs. (**A**) Percentages of CD1a^+^ CD14^−/lo^, CD1a^hi^ CD14^−/lo^ and CD1a^−^ CD14^+^ cells recovered after 5–6 days of culture in the presence of conditioned media from control (grey bars; CM-DCs) and BMP4-transduced (black bars; BMP/CM-DCs) ALL cells. Data represent the mean ± SEM of 10 to 12 independent experiments. (**B**) Representative histograms showing CD1a expression in CM- and BMP/CM-DCs. Percentages of CD1a^hi^ cells are shown. For comparison, red line shows CD1a expression in DCs grown in the absence of conditioned media. (**C**) Real-time PCR quantification of mRNA levels in DCs differentiated from monocytes in the presence of conditioned media from control (grey bars) and BMP4-transduced (black bars) ALL cells. Relative mRNA expression was calculated by dividing all individual data by the mean expression in CM-DCs. Results represent the mean ± SEM of three to six independent experiments. (**D**) Histograms show the percentages of proliferating CD4^+^ and CD8^+^ T cells, gated on the CD3^+^ cell population and calculated by the CFSE dilution method in mixed lymphocyte reaction assays. Data are the mean ± SEM of four to six independent experiments. Supernatants from DC/T cell co-cultures were harvested at day 5–6 and the amount of IFN-γ was quantified by ELISA. Data are the mean ± SEM of five to six independent experiments. Asterisks represent statistically significant differences between CM-DCs and BMP/CM-DCs (* *p* ≤ 0.05; by Mann–Whitney test).

Conditioned media from BMP4-overexpressing ALL cells (BMP/CM) (Table 2) was next used to generate DCs from monocytes (BMP/CM-DCs). In the presence of BMP/CM the proportion of CD1a^+^ CD14^−/lo^ cells was further reduced when compared to CM-DCs, being the terminally differentiated CD1a^hi^ CD14^−/lo^ DC subset the most affected by the high BMP4 expression (Figure 2A,B). A parallel increment in the proportion of CD1a^−^ CD14^+^ cells was also observed in the presence of BMP/CM (Figure 2A).

Gene expression analysis showed that, in comparison to CM-DCs, BMP/CM-DCs expressed higher levels of IL-10, and also TNF-α (Figure 2C), what hardly affected the low TNF-α/IL-10 expression ratio. The presence of high levels of BMP4 during DC differentiation also induced an increased expression of TGF-β, IL-6 and mainly IL-1β and IL-8 (2- to 4-fold increases) (Figure 2C). Upregulated transcription levels of IDO1 and MMP9 were also detected in BMP/CM-DCs (Figure 2C).

In agreement with the acquisition of a more pronounced immunosuppressive phenotype, the co-culture with CD3^+^ T lymphocytes evidenced that BMP/CM-DCs exhibited a diminished allostimulatory capacity (Figure 2D and Supplementary Figure S2). The proliferative response of CD8^+^ T cells was much less affected than that of CD4^+^ T cells, and consequently IFN-γ secretion underwent only a further slight decrease when compared with CM-DCs (Figure 2D).

### 3.3. ALL Cells Promote MØ Polarization Towards an Anti-inflammatory M2-Like Phenotype

**Figure 3:**
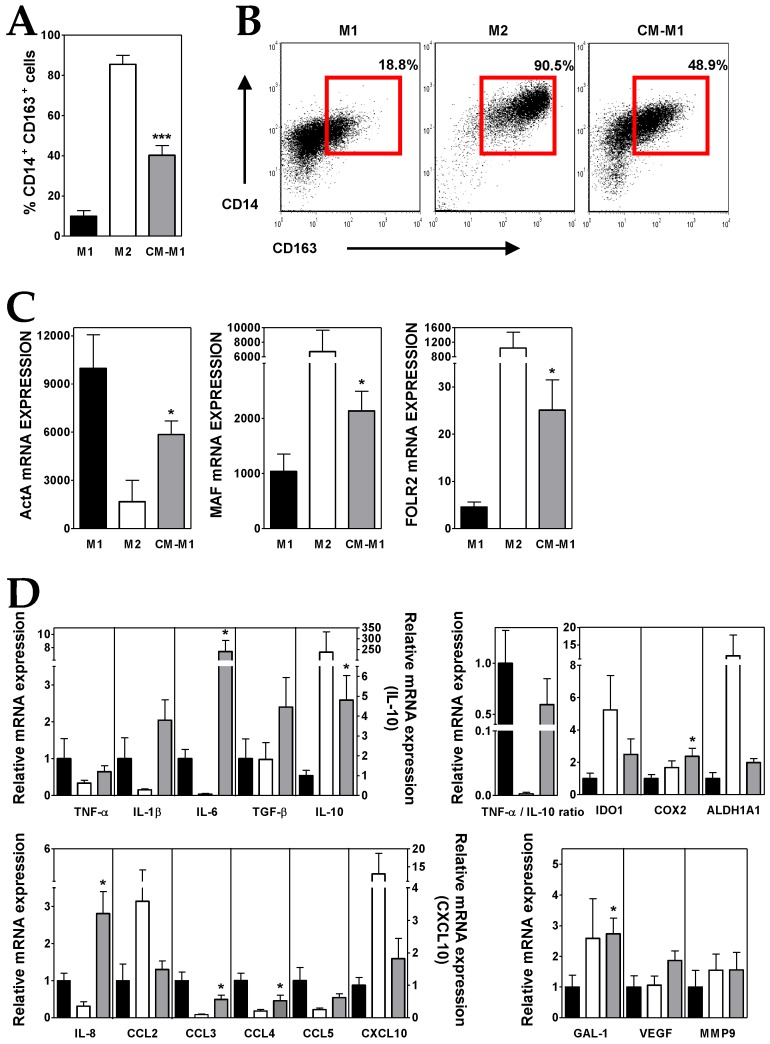
ALL cells promote M2-like macrophage (MØ) differentiation. (**A**) Percentages of CD14^+^ CD163^+^ MØs generated from monocytes cultured for five days with GM-CSF (black bars; M1), M-CSF (white bars, M2) and GM-CSF plus conditioned media from ALL cells (grey bars; CM-M1). Data represent the mean ± SEM of 12 independent experiments. (**B**) Representative dot plots showing CD14 versus CD163 expression. Percentages of CD14^+^ CD163^+^ MØs, delimited by red gates, are shown. (**C**,**D**) Real-time PCR quantification of mRNA levels in MØs differentiated from monocytes after 3 days of culture with GM-CSF, M-CSF and GM-CSF plus conditioned media from ALL cells. Relative mRNA expression in (**D**) was calculated by dividing all individual data by the mean expression in M1 MØs. Results represent the mean ± SEM of three to ten independent experiments. Asterisks represent statistically significant differences between M1 and CM-M1 MØs (* *p* ≤ 0.05 and *** *p* ≤ 0.001; by Mann–Whitney test).

Peripheral blood monocytes were cultured with rhGM-CSF, in the absence or presence of ALL-derived CM, to induce the differentiation to pro-inflammatory M1-like MØs. For comparison, anti-inflammatory M2-like MØs were differentiated from monocytes by culturing them with rhM-CSF. The addition of ALL-derived CM during M1 differentiation induced an average 4-fold increase in the proportion of CD14^+^ CD163^+^ cells, a phenotype usually associated with M2 features in MØs [29] (Figure 3A,B). The analysis of the expression of several markers differentially expressed by M1- and M2-like MØs [30] showed that ALL-derived CM caused a notable reduction (~50%) in the expression of the M1 marker Activin A (ActA) (Figure 3C). In addition, the presence of ALL-derived CM induced the expression of the M2 markers folate receptor β (FOLR2) and MAF transcription factor (5- and 2-fold increases, respectively) (Figure 3C). Intermediate expression levels of CD206, CD209 and HLA-DR were also observed (Supplementary Figure S1B). These observations indicated that some factors present in ALL-derived CM skewed M1-like MØ polarization towards a less pro-inflammatory phenotype. Interestingly, the neutralization of BMP4 in ALL-derived CM reduced (~40–50%) the generation of CD14^+^ CD163^+^ cells and partially inhibited the increases in FOLR2 and MAF expression and the reduction in ActA expression (data not shown; manuscript in preparation).

A further characterization supported that the culture of monocytes with ALL-derived CM during M1 polarization induced the acquisition of an immunosuppressive profile in the resulting MØs (CM-M1). Compared to M1, CM-M1 MØs exhibited a decreased expression of TNF-α and an upregulated IL-10 expression (Figure 3D), what reduced by 40% the TNF-α/IL-10 expression ratio. Likewise, CM-M1 MØs showed intermediate features between M1- and M2-like MØs for the expression of the chemokines CCL2, CCL3, CCL4, CCL5 and CXCL10 as well as IDO1 and ALDH1A enzymes (Figure 3D). By contrast, the transcription of IL-6 and IL-8, but also IL-1β, TGF-β, COX2, GAL-1, VEGF and MMP9, was notably upregulated in CM-M1 MØs, even above the M1 and M2 values (Figure 3D).

### 3.4. BMP4 Overexpressing-ALL Cells Favour the Generation of M2-Like MØs with Pro-tumoural Features 

**Figure 4:**
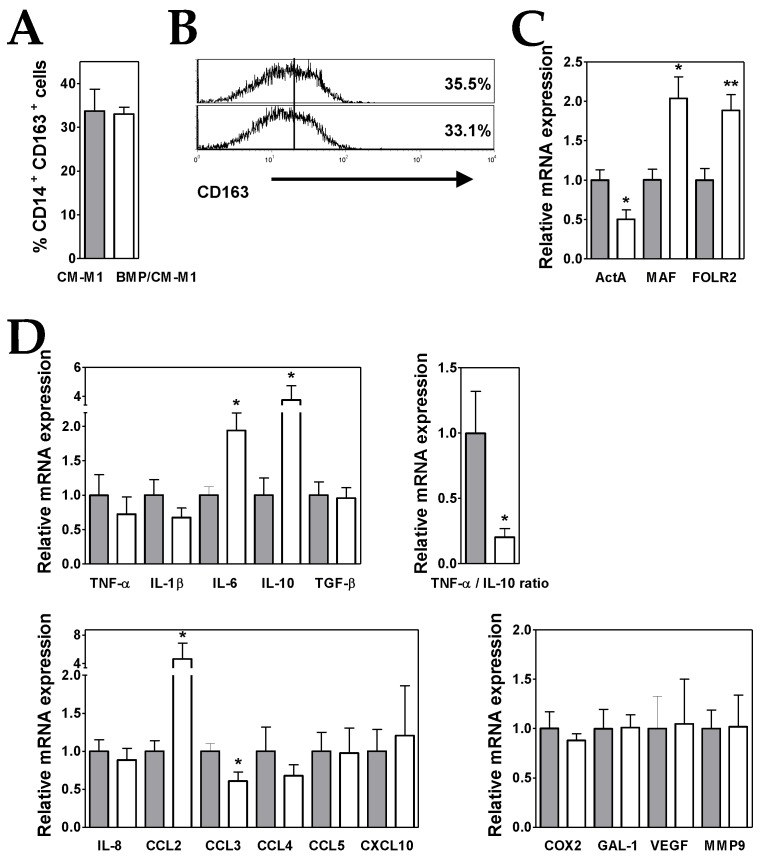
BMP4 overexpression in ALL cells potentiates their ability to generate M2-like MØs. (**A**) Percentages of CD14^+^ CD163^+^ MØs generated from monocytes cultured for five days with GM-CSF plus conditioned media from control (grey bars; CM-M1) and BMP4-transduced (white bars; BMP/CM-M1) ALL cells. Data represent the mean ± SEM of four independent experiments. (**B**) Representative histograms showing CD163 expression in CM-M1 and BMP/CM-M1 MØs. (**C**,**D**) Real-time PCR quantification of mRNA levels in MØs differentiated from monocytes after three days of culture with GM-CSF plus conditioned media from control (grey bars) and BMP4-transduced (white bars) ALL cells. Relative mRNA expression was calculated by dividing all individual data by the mean expression in CM-M1 MØs. Results represent the mean ± SEM of three to five independent experiments. Asterisks represent statistically significant differences between CM-M1 and BMP/CM-M1 MØs (* *p* ≤ 0.05 and ** *p* ≤ 0.01; by Mann–Whitney test).

Monocytes were induced to differentiate into M1-like MØs in the presence of conditioned media from BMP4-overexpressing ALL cells (BMP/CM-M1). Under high levels of BMP4, similar proportions of CD14^+^ CD163^+^ cells were generated (Figure 4A,B) although BMP/CM-M1 MØs displayed an enhanced M2-like phenotype, in comparison to CM-M1 MØs. Expression levels of M2 markers MAF and FOLR2 underwent a further 2-fold increase whereas ActA transcription was additionally repressed by 50% (Figure 4C).

When compared to CM-M1 MØs, the gene expression analysis showed that BMP/CM-M1 MØs also exhibited a less pro-inflammatory profile though with some enhanced pro-tumoural features (Figure 4D). A 4-fold increment in IL-10 expression together with a reduced TNF-α expression led to a remarkable further decrease in the TNF-α/IL-10 expression ratio (by about 80%) (Figure 4D). In addition, the expression levels of CCL2 and IL-6 were upregulated (4- and 2-fold increases, respectively) in comparison to CM-M1 MØs (Figure 4D).

## 4. Discussion

Immune evasion is a recognized hallmark of cancer, being the inhibition of the normal anti-tumour functions of DCs and MØs one of the immunosuppressive mechanisms used by cancer cells to evade immunity in solid as well as haematological malignancies. 

Quantitative and qualitative alterations in DCs have been described to be a common feature shared by different haematological tumours including chronic myeloid leukaemia [31], myelodysplastic syndromes [32], acute myeloid leukaemia [33], multiple myeloma [34] or chronic lymphocytic leukaemia [35]. Similarly, in B cell precursor ALL patients the levels of both conventional and plasmacytoid DCs were reduced in blood as well as in bone marrow at diagnosis, and DC levels were related to the extent of the disease being lower in those patients with unfavourable prognostic features [16,36,37]. Likewise, Zhou et al. have described an aberrant functionality of DCs in adult B lineage ALL [38] and Mami et al. [37] were not able to generate CD1a^+^ myeloid or ILT3^+^ plasmacytoid DCs from circulating CD34^+^ precursor cells of ALL patients, suggesting that DC differentiation was altered in B cell precursor ALL. In support, our data provide evidence that DCs differentiated from monocytes in the presence of ALL-derived soluble factors show an atypical phenotype exhibiting features usually seen in tolerogenic DCs as well as immunosuppressive tumour-associated DCs [7,39,40,41]. These characteristics include a low TNF-α/IL-10 expression ratio and high expression of the immunosuppressive cytokine TGF-β and other cytokines (IL-6, IL-1β), chemokines (mainly CCL2, CCL5 and IL-8) and factors (the COX2 and ALDH1A enzymes involved in biosynthesis of prostaglandins and retinoic acid, respectively, the pro-angiogenic factor VEGF, and the protease MMP9 engaged in degradation and remodelling of extracellular matrix). All in combination would contribute to impair the effector immune response, to favour monocyte recruitment and the development of other cell types with immunosuppressive activity and to promote the growth, survival and invasiveness of leukemic cells. 

In haematological malignancies, as in solid tumours, MØs also infiltrate the tumour tissues and many of these tumour-associated MØs are induced to differentiate to M2-like MØs exhibiting pro-tumoural functions [9,42]. The frequency of CD163^+^ M2-like MØs has been reported to be notably increased in acute and chronic myeloid leukaemia [43,44], chronic lymphocytic leukaemia [45], multiple myeloma [46] and also adult ALL [47]. In line with these data, our results show that soluble factors derived from ALL blasts are able to skew the differentiation of monocytes cultured under M1 conditions towards a M2-like phenotype. These MØs express CD163 and M2-specific markers (increased expression of FOLR2 and MAF and reduced expression of ActA), and show an upregulated expression of different factors that favour tumour progression (including, among others, IL-10, TGF-β, IL-6, IL-8, IDO1, COX2 and GAL-1). 

The generation of immunosuppressive/tolerogenic DCs and M2 MØs has been proposed to be induced by different tumour-mediated mechanisms such as hypoxia, endoplasmic reticulum stress, and mainly exposition to several tumour-derived cytokines and growth factors [6,8,13,48]. In this context, the components of the TGF-β superfamily, including the BMPs, have been extensively reported to participate in multiple aspects of tumour biology including immune evasion [21,49,50,51] but the relevance of BMP pathway in the origin and progression of leukaemias and lymphomas is now beginning to be uncovered [24,52]. Our present data show that Nalm-6 ALL cells mainly secrete BMP4, but not other BMP ligands previously reported to be involved in lymphoid malignancies [24,53]. In other ongoing studies, we also described the expression of BMP4 in primary ALL blasts obtained at the time of diagnosis, and notably this BMP4 expression was significantly increased in ALL cells derived from paediatric patients who later relapsed [54] (manuscript in preparation). In agreement with our results, Gaynes et al. [25] reported an upregulated expression of BMP4 in ALL cells infiltrating the central nervous system of transplanted NSG mice, in comparison to those leukemic cells located in bone marrow. However, Tesfai et al. [26] only found a BMP2 overexpression when compared pre-B ALL and CD34^+^ cells.

We also point out that BMP4-containing CM from ALL cells impair the differentiation of DCs and MØs from monocytes. The relevance of BMP4 is further supported by the results showing that BMP4 overexpression in ALL cells enhances the generation of immunosuppressive DCs which in addition exhibit a higher pro-tumoural activity, revealed by an upregulated expression of the immunosuppressive and tumour growth promoting factors TGF-β, IL-6, IL-1β, IL-8, IDO1 and MMP9, and a concomitant reduced allostimulatory capacity. The involvement of BMP signalling in DC differentiation and maturation has been previously reported by us and others [55,56]. BMP4-overexpressing ALL cells also have the ability to generate MØs with a more marked M2-like phenotype and enhanced pro-tumoral features evidenced by a lower TNF-α/IL-10 expression ratio and upregulated expression levels of CCL2 and IL-6. In this line, we have recently described that BMP4, produced by bladder cancer cells, induces monocyte differentiation toward a M2 phenotype, leading to the production of cytokines that favour tumour progression [57]. Interestingly, the ability to induce M2 macrophage polarization seems to be shared with other BMP ligands since BMP2 promotes the acquisition of a M2 phenotype during bone regeneration [58] and BMP-7 treatment increases M2 differentiation and reduces inflammation and plaque formation in atherosclerosis [59].

Together, our results indicate that BMP4 is an important ALL blast-derived soluble factor which contribute to switch the differentiation of monocyte-derived DCs from an immunostimulatory to an immunosuppressive state, and to promote the polarization of MØs to a pro-tumoural phenotype. Future work could focus on the blockade of BMP4 secretion by ALL cells which could help, in those patients who relapse, to control leukaemia progression by counteracting the development of a pro-tumour immune microenvironment.

## Figures and Tables

**Table 1:** TaqMan gene expression assays.

Gene	Reference	Gene	Reference
TNF-α	Hs00174128_m1	COX2	Hs00153133_m1
IL-1β	Hs01555410_m1	ALDH1A1	Hs00946916_m1
IL-6	Hs00985639_m1	GAL-1	Hs00355202_m1
IL-10	Hs00961622_m1	VEGF-A	Hs00900055_m1
TFG-β1	Hs00998133_m1	MMP9	HS00234579_m1
IL-8	Hs00174103_m1	FOLR2	Hs_01044732_g1
CCL2	Hs00234140_m1	MAF	Hs_04185012_s1
CCL3	Hs00234142_m1	ACTA	Hs01081598_m1
CCL4	Hs00237011_m1	BMP2	Hs00154192_m1
CCL5	Hs00174575_m1	BMP4	Hs00370078_m1
CXCL10	Hs01124251_g1	BMP6	Hs01099594_m1
IDO1	Hs00984148_m1	BMP7	Hs00233476_m1

**Table 2:** Expression of Bone Morphogenetic Protein (BMP) ligands in acute lymphoblastic leukaemia (ALL) cell lines.

	Control Nalm-6	BMP4-transduced Nalm-6
BMP2 mRNA Expression (arbitrary units)	ND	ND
BMP4 mRNA Expression (arbitrary units)	44 ± 15	43384 ± 4260
BMP6 mRNA Expression (arbitrary units)	ND	ND
BMP7 mRNA Expression (arbitrary units)	ND	ND
BMP4 Expression (pg/mL)	7 ± 1	1786 ± 35

ND: Not detected/Under the limit of detection.

## Supplementary Materials

The following are available online at https://www.mdpi.com/2073-4409/8/7/722/s1, Figure S1: Phenotypic analysis of DCs and MØs differentiated in the absence or presence of conditioned media from ALL cells, Figure S2: Representative histograms showing the allostimulatory capacity of DCs differentiated in the absence or presence of conditioned media from ALL cells, Figure S3: Conditioned media from different ALL cell lines alter the differentiation of DCs and MØs.
