# Supplementary material for: Acute Lymphoblastic Leukaemia Cells Impair Dendritic Cell and Macrophage Differentiation: Role of BMP4
Source: Cells. 2019 Jul 14;8(7):722. doi: 10.3390/cells8070722 (PMC6679123; doi:10.3390/cells8070722)
Supplement: Supplementary file 1 [file cells-08-00722-s001.pdf]

# Figure S1

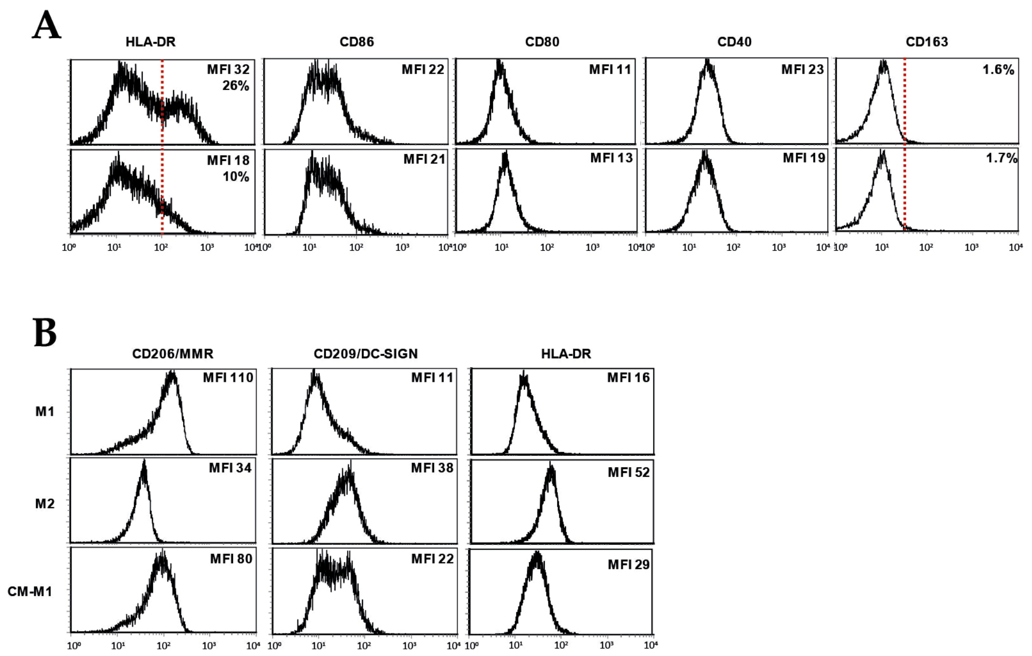


**Figure S1**: Phenotypic analysis of DCs (A) and MØs (B) differentiated in the absence or presence of conditioned media from ALL cells. Histograms show the expression of several surface molecules analysed by flow cytometry. The mean fluorescence intensity (MFI) of the whole cell population is shown in the histograms. The percentage of HLA-DR$^{high}$ and CD163$^{+}$ DCs is also included in the corresponding histograms.

# Figure S2

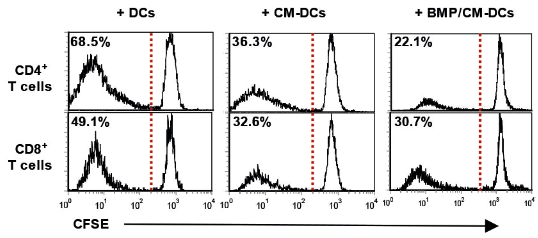


**Figure S2:** Allostimulatory capacity of DCs differentiated in the absence or presence of conditioned media from ALL cells. Representative histograms show the percentages of proliferating (CFSE-low) $CD4^+$ and $CD8^+$ T cells.

# Figure S3

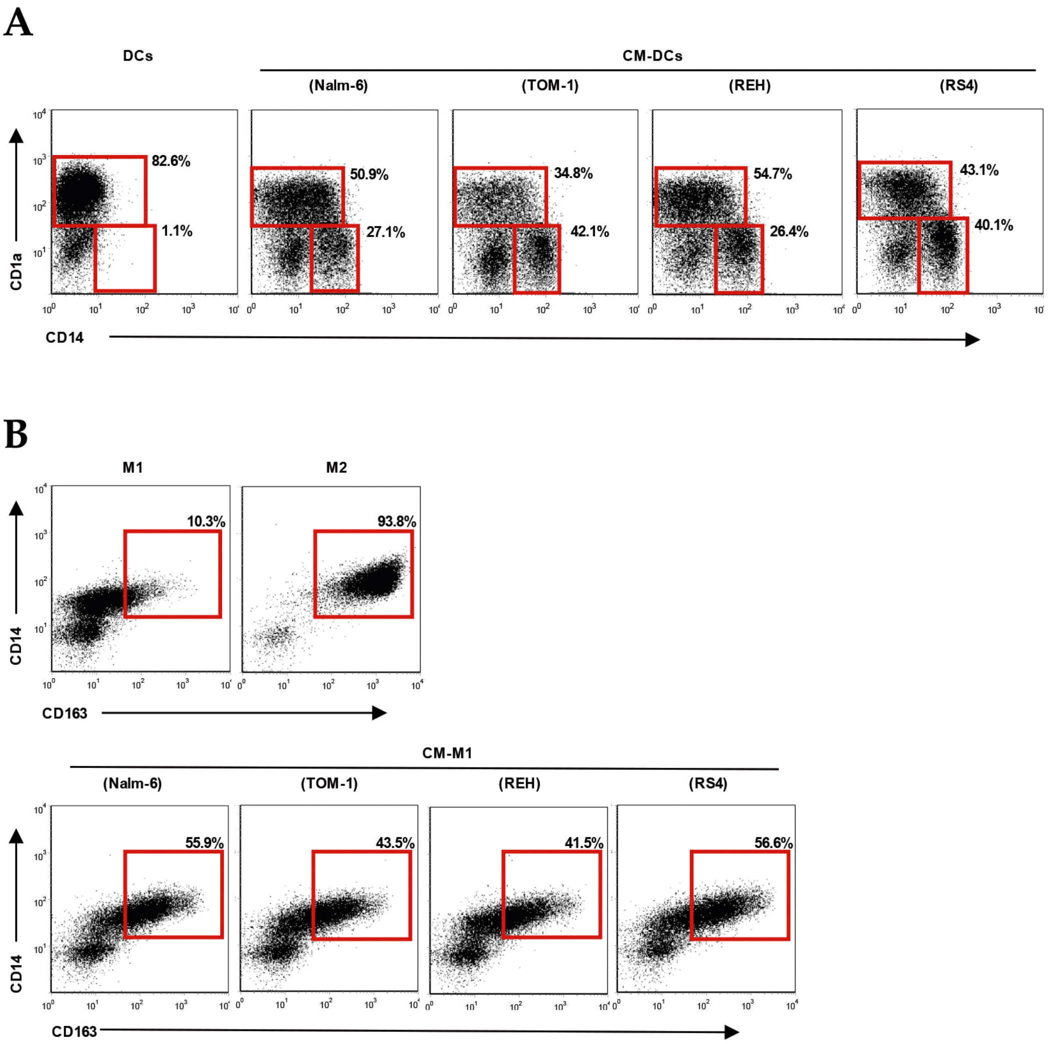


**Figure S3:** Conditioned media from different ALL cell lines alter the differentiation of DCs and MØs. (A) Representative dot plots show CD14 versus CD1a expression in DCs, and the percentages of CD1a$^{+}$ CD14$^{-/lo}$ and CD1a$^{-}$ CD14$^{+}$ cell populations, delimited by red gates, are shown. (B) Representative dot plots show CD14 versus CD163 expression in MØs, and the percentages of CD14$^{+}$ CD163$^{+}$ cells, delimited by red gates, are shown.
